# Spatial Learning Is Impaired in Male Pubertal Rats Following Neonatal Daily but Not Randomly Spaced Maternal Deprivation

**DOI:** 10.3389/fcell.2021.621308

**Published:** 2021-03-18

**Authors:** Emily T. Stoneham, Daniel G. McHail, Sabina Samipour-Biel, Nicole Liehr, Christina M. Lee, Jean C. Evans, Katelyn Boggs, Theodore C. Dumas

**Affiliations:** ^1^Krasnow Institute for Advanced Studies, George Mason University, Fairfax, VA, United States; ^2^University of Illinois, Champaign, IL, United States; ^3^George Mason University, Fairfax, VA, United States; ^4^INOVA Fair Oaks Hospital, Fairfax, VA, United States; ^5^Virginia Tech, Blacksburg, VA, United States

**Keywords:** brain derived neuronal factor, corticosterone, hippocampus, glucocorticoid receptor, maternal deprivation, tyrosine receptor kinase B, spatial learning

## Abstract

Severe early life stress has long been associated with neuropsychological disorders in adulthood, including depression, schizophrenia, post-traumatic stress disorder, and memory dysfunction. To some extent, all of these conditions involve dysregulation of the hypothalamic-pituitary-adrenal (HPA) axis and reduced negative feedback inhibition of cortisol release in adulthood. However, the time course for mental health and hormonal outcomes across life stages and the attributes of early life stress that direct the behavioral and biological alterations is not fully understood. We designed our studies to compare outcomes of the two most common maternal deprivation schedules on cognitive ability prior to adulthood. We exposed rat pups to daily or randomly spaced maternal separation bouts within the first 3 weeks of life and examined cognitive performance, neurotrophic signaling, and stress and immune system markers during puberty. We found that the daily separation schedule impaired spatial learning while the randomly spaced schedule did not alter maze performance relative to normally reared control animals. Animals that underwent daily separation showed a tendency for reduced body weight compared to the randomly spaced condition, but there were no differences in adrenal weight. Thymus weight normalized by body weight was increased following daily separation compared to random separation and control conditions. Plasma corticosterone levels measured after behavior testing did not differ amongst experimental groups and there was no impact of TrKB receptor inhibition. Combined, the results show that different early life stress schedules produce different behavioral and biological outcomes when measured at puberty. Combined with prior findings from more mature animals, the results presented here suggest that daily neonatal stress produces varied alterations in spatial cognition at different life stages with a transient learning deficit at puberty preceding a more persistent and a progressive memory impairment through adulthood and into aging.

## Introduction

Survivors of early life abuse frequently suffer neuropsychological disorders in adulthood, including depression, schizophrenia, post-traumatic stress disorder, and memory dysfunction. All of these conditions are linked to dysregulation of the hormonal stress response, the hypothalamic-pituitary-adrenal (HPA) axis, with depression and memory loss more specifically associated with chronic increases in serum cortisol (corticosterone in rodents, abbreviated CORT). Maternal deprivation (MD) in animal models supports the notion that early life stress sets up a positive feedback loop whereby lasting elevations in CORT during development impair systems that regulate CORT release later in life (Stanton et al., 1988; Champagne, 2008). These cognitive and HPA impairments can result, in part, from atrophy and impaired function of the hippocampus (Huot et al., 2002; Li et al., 2010). Specifically, dendritic atrophy, disrupted synaptic plasticity, reduced neurogenesis (Karten et al., 2005), and progressive neuron loss (Brunson et al., 2005).

Rodent MD involves removing rat pups from the home cage for varying numbers of hours per day and varying numbers of days during the first three postnatal weeks, with outcomes measured in adulthood (Meaney et al., 1991; Plotsky and Meaney, 1993; van Oers, 1998; Aisa et al., 2007; Daskalakis et al., 2011; Suri et al., 2013). Many early life stress studies in rats can be more broadly classified as one of two MD paradigms, daily or intermittent/pseudo-random (Tractenberg et al., 2016). In the daily MD model, separation occurs every day, typically during the first postnatal week (Plotsky and Meaney, 1993; Kioukia-Fougia, 2002; Weaver et al., 2007). In this model, consistent findings include reductions in hippocampal expression levels for the glucocorticoid receptor (GR) (Ladd et al., 2004; Seo et al., 2020) and the neuroprotective factor, brain derived neurotrophic factor (BDNF) (Seo et al., 2016; Park et al., 2018), accompanied by hippocampal atrophy (Sapolsky, 1987), adrenal hyperplasia (Ulrich-Lai et al., 2006; Herman et al., 2016), excessive CORT release (Ulrich-Lai et al., 2006; Herman et al., 2016), thymus atrophy (Ulrich-Lai et al., 2006; Herman et al., 2016), and a long-term spatial memory deficit in adulthood (Brunson et al., 2005; Li et al., 2006; Herman et al., 2016). For the intermittent/pseudo-randomly spaced MD model, separation is not daily and does not occur at regular intervals (van Oers, 1998; Lehmann et al., 1999; Weaver et al., 2007). In this model, reductions in basal CORT (Kuhn and Schanberg, 1998; Daskalakis et al., 2011), increased sensitivity to glucocorticoid negative feedback (Kuhn and Schanberg, 1998; De Kloet et al., 2005; Daskalakis et al., 2011), increased adaptability to social settings (De Kloet et al., 2005; Oomen et al., 2010), and improved emotional resilience are evident (Price and Steckler, 2011; Schmidt et al., 2011). Because these different separation schedules have been employed independently, contrasting reports could result from any number of undocumented variables. Moreover, prior work has focused on outcome in middle aged and older rats, preventing a more complete understanding of the progressive alterations to immune, hormonal, and neural processes that lead to adult memory loss.

The reduced negative feedback of CORT release following daily MD stems from hypermethylation of the neuron-specific GR promoter, *NR3C* (Weaver et al., 2004), and a reduction in the hippocampal GRs (Anisman et al., 1998). A loss of hippocampal GRs and reduced negative feedback regulation of circulating CORT promotes synaptic depression, hippocampal atrophy, memory impairment, and psychological depression (McEwen et al., 1997; Kim and Diamond, 2002; Xiong and Krugers, 2015). Additionally, daily MD produces a reduction in the expression of BDNF in adulthood (Lippmann et al., 2007; Chourbaji et al., 2010; O’Sullivan et al., 2011; Lee et al., 2012). Impaired BDNF signaling results in a loss of a critical factor supporting late phase long-term potentiation of synaptic efficacy, dendritic growth, neurogenesis, and long-term memory (McEwen et al., 2015). Reductions in hippocampal GR and BDNF levels due to early life stress likely increase susceptibility to hippocampal related impairments later in life (Heim et al., 2004; Suri et al., 2013) and highly similar biological findings have emerged from human studies (Caffey and Silbey, 1960; Bonanno et al., 2002). A better understanding of alterations in CORT and BDNF signaling through adolescence might bring greater clarity to how the adult cognitive disability emerges and could reveal biological markers of impending memory failure.

The few experiments that examined effects of MD at varying testing ages have shown that impacts on cognitive ability are not immediately observed (Workel et al., 2001; Pardon and Rattray, 2008; Nishi et al., 2013) and do not emerge until well into adulthood (Brunson et al., 2005; Zalosnik et al., 2014; Shu et al., 2015). The delayed onset of symptoms supports the notion that most, if not all of the health problems produced by early life stress may be substantially alleviated with targeted prophylactic therapy through identification of behavioral and biological markers in adolescents and young adults. In an attempt to discover early behavioral and biological markers for impending memory impairment and to better understand the impact of stress schedule, we applied daily or randomly spaced MD to neonatal rats and measured outcomes at puberty and compared behavioral, hormonal, immune, and health outcomes. We discovered that daily MD impaired spatial learning, while randomly spaced MD had no effect. Interestingly, while behavioral differences were observed, no alterations in plasma CORT levels were observed after behavior testing. Adrenal and thymus weights were unaltered, but body weight tended to be reduced following daily MD. These results further dissociate the daily and randomly spaced MD paradigms and reveal a potential behavioral marker in pubertal subjects for impending memory dysfunction in adulthood as a result of early life stress.

## Materials and Methods

### Subjects

Long-Evans hooded rats bred in-house were for this study. Breeder males were obtained from Charles River and breeder females were bred in-house. Male offspring were used as test subjects because puberty related changes in the levels of reproductive hormones known to modulate hippocampal anatomy (Woolley and McEwen, 1993) and spatial cognition (Berry et al., 1997; Warren and Juraska, 1997; Korol et al., 2004) occur over a shorter timespan in female than in male rats (Ojeda and Skinner, 2006) and overlap to a greater extent with the experimental manipulations and test. Female offspring remained in the home cage with the dam at all times to buffer the dam from the stress of pup removal. Also, only the first litter from each dam was used for this study to control for effects of maternal history on dam-pup interactions. Day of birth was designated as postnatal day (P) 0. Litters were culled to 12 pups on P1, retaining even numbers of male and female rats when possible and litters of less than six pups were not used. Whole litters were randomly assigned to either MD condition or control on P1.

All animals were housed in individually ventilated laboratory rat cages (height: 20.3 cm; length: 30.5 cm; width: 35.5), under a 12 h light/dark cycle (lights on at 7:00 a.m.), and in a breeding colony with controlled temperature (23 ± 2°C) and humidity (30–70% relative humidity). Fresh food pellets (7012 Harlan Teklad LM-485, Fredrick, MD) and drinking water were available *ad libitum.* All experiments were conducted in accordance with the guidelines specified by the National Institutes of Health (*NIH Guide for the Care and Use of Laboratory Animals;* (NIH Publications No. 8023, revised 1978) and approved by the Institutional Animal Care and Use Committee of George Mason University.

**FIGURE 1:**
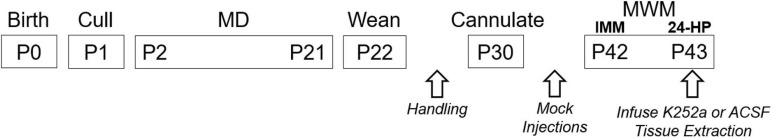
Event timeline. The onsets and durations for all experimental events are depicted. All animals were handled briefly on P23–28. Mock injections were performed on P34–39. Drug (K252a) or ACSF was infused 1 h prior to the 24-HP. Tissues were harvested after the 24-HP. IMM, Immediate probe trial performed on the same day as training.

Figure 1 summarizes the timeline for all experimental procedures. Following MD, male and female offspring were weaned at P22. From this point forward, females were group housed and males were individually housed. Test subjects were handled between MD and cannulation surgery at P30 followed by mock injections following cannulation surgery to desensitize the animals to the cannulation restraint prior to behavior testing. At P42, testing in the Morris water maze (MWM) ensued and drug (K252a, tyrosine receptor kinase B (TrKB) inhibitor) or vehicle was delivered directly to the hippocampus on the final day prior to the long-term memory test (the 24 h probe trial, 24-HP). Following behavior testing, subjects were euthanized and tissues were harvested. Each individual event is described below.

### Maternal Deprivation (MD)

Test subjects were separated from the dam according to two schedules. For daily MD (labeled P2–11), animals were separated from the dam once per day for ten consecutive days from P2 to P11. The random assignment group (labeled RAN) experienced the same number of separation days but spaced from P2 to P20. MD days for the RAN litters were determined by an on-line random number generator (random.org). Additional requirements for the randomization were that at least three of the separation days fall between P2 and P7 and that there were no more than three consecutive separation days anywhere on the schedule. The control litters (labeled CON) were left completely undisturbed until weaning except for a single cage change by the experimenter at ∼P12.

On the first day of MD, the dam from a single litter was removed to a separate transfer cage, male pups were removed to a different transfer cage, and female pups were removed to a new, clean temporary home cage before the dam was reunited with the female pups in the new cage. The dam and female pups remained in the housing room, while the male pups were brought to an adjacent room and individually placed in plexiglass isolation chambers without bedding (15 cm L × 15 cm W × 10 cm H) for 3 h per session. For pups in the RAN group, MD that occurred after P14 required the use of clear, plexiglass cage covers to prevent escape. Pups in all separation groups were warmed with an infrared heat lamp placed over the isolation chambers. The side of the chamber closest to the lamp was maintained at 32°C, the middle of the chamber at 30°C and the farthest point at 28°C. To mask vivarium noise and the sounds of the other pups, white noise was played through speakers at 75–80 dB as verified by an iPhone digital noise meter (Decibel Meter Pro). At the end of the 3 h period, male pups were put back into their transfer cage and returned to the housing room. The dam was moved to a temporary transfer cage while male pups were moved back to the permanent home cage at which time the dam was returned to the permanent home cage. After weaning on P22, male pups were singly housed and handled daily for 3 min per day on P23–28.

### Surgery

**FIGURE 2:**
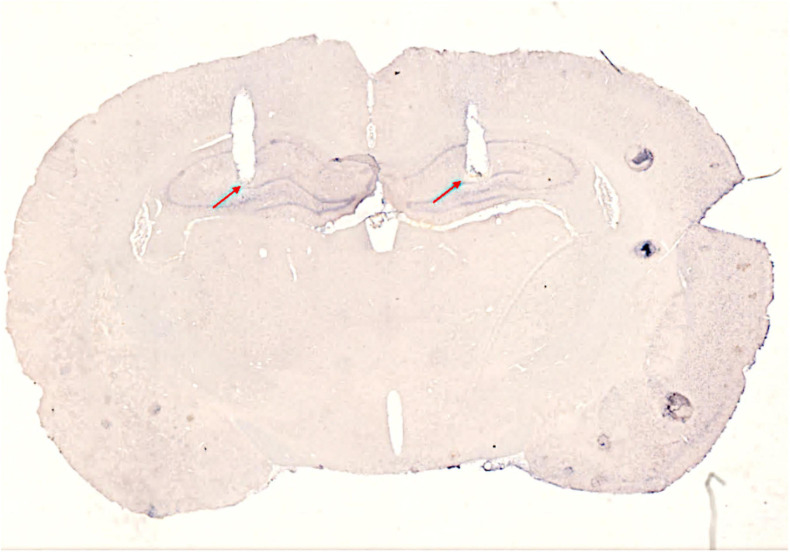
Image of a sagittal brain section showing cannula placement. Red arrows designate the most ventral extents of the cannulae tracts. The triangular notch in the right hemisphere designates brain orientation.

At P30, animals underwent stereotaxic surgery under Isoflurane anesthesia to implant bilateral indwelling guide cannulae. A mid-sagittal incision approximately 1.5 cm in length was made and the wound was held open with locking forceps. The fascia was scraped from the skull with the sharp edge of a pair of forceps and the skull was sterilized with hydrogen peroxide (3%) and ethyl alcohol (70%). Three small screws (MX-0080-02F-C, Small Parts, Amazon Inc.) were inserted into burr holes positioned around the intended cannula insertion zones to serve as anchors. Two burr holes were drilled at 3.6 mm posterior from bregma and ± 2.5 mm from the midline. One cannula (stainless steel hypodermic tubing, 26ga, Small Parts, Miramar, FL) was lowered into each hole simultaneously to 3.0 mm below dura so that the tips of both cannulae sat approximately 1 mm above the apex of the DG in both hippocampi (Dumas et al., 2010). Figure 2 shows a thin section from the brain of an animal with bilateral cannulae implants. Stylets extended approximately 0.5 mm beyond the tips of the cannulae and were left in place until mock injections were performed. Cannulae were cemented into place with dental cement (Grip Cement, Dentsply International Inc., Milford, DE). Once the dental cement had set, the scalp wound was clipped closed around the cement headcap and treated with topical antibiotic (bacitracin, Neosporin, Johnson & Johnson, New Brunswick, New Jersey). Rats were weighed prior to surgery and their tails were tattooed with a unique identifier during the surgery. Post-surgery, rats were given ketoprofen (5 mg/kg) subcutaneously two times a day for 3 days. Rats were allowed 10 days to recover from surgery prior to behavior testing. From P34 to P39, animals underwent brief head restraint and mock cannula insertions.

### Drug Delivery

The selective tyrosine receptor kinase B (TrKB) inhibitor, K252a (Sigma Aldrich, St. Louis, MO) was diluted to 31 ng/μl in artificial cerebral spinal fluid (ACSF in mM: NaCl 124, KCl 2, MgSO_4_ 2, CaCl_2_ 2, KH_2_PO_4_ 1.25, NaHCO_3_ 26, and glucose 10, pH 7.4). This drug and dose were chosen to match other BDNF signaling and memory studies using rats of nearly the same age (Whitfield et al., 2011; Wang et al., 2012; Ju et al., 2015). One hour prior to the 24-HP probe (on P43), animals were infused with either K252a or artificial cerebrospinal fluid (ACSF) at a rate of 0.3 μL per minute for a total of 0.8 μL per hemisphere. Canulae remained in place for 2 min following infusion to optimize drug diffusion.

### Morris Water Maze Testing

**FIGURE 3:**
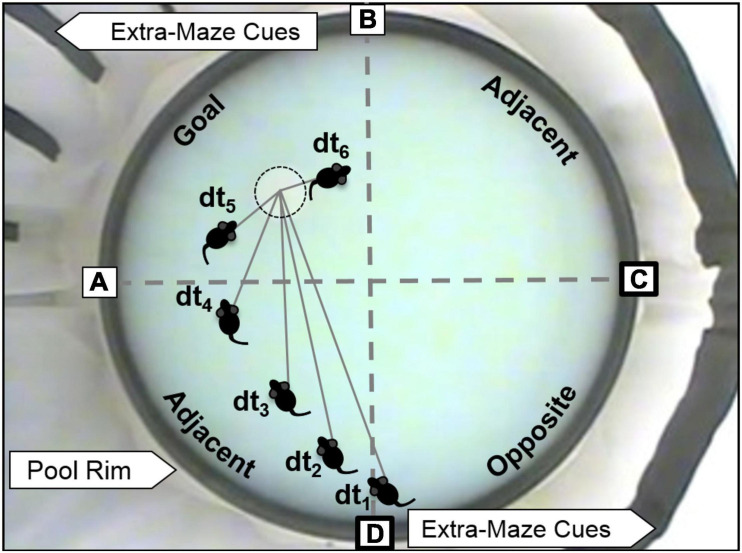
MWM and depiction of spatial analyses. The escape platform is just barely visible in the Goal quadrant (with dotted circle). Spatial cues can be seen attached to curtains that surround the pool. Boxed letters indicate the pseudo-randomized starting locations used during training and probe trials. Bold boxes indicate potential start locations for probe trials for the platform location shown in the figure. Dashed gray lines indicate the boundaries of the four quadrants (Goal, two Adjacent, and Opposite). Distance to platform (DtP) analysis is conducted by noting the linear distance between the center of the rat and the platform center (D) in video snapshots separated by 1 s (t_x_).

The Morris water maze (MWM) was used to test spatial learning and memory. A black circular pool (1.7 m in diameter) was filled with tap water (23–24°C) to 10 cm below the rim of the pool, a level that covered a stationary escape platform (17.5 cm diameter) by 1 cm. White curtains displaying black spatial cues surrounded the pool (Figure 3). MWM procedures were followed as described previously (Dumas et al., 2010) except that the water was made opaque with non-toxic white paint and the escape platform was covered with white, non-slip, rubber shelf-liner. A video camera (Model WV-CP280-N, 1/3″ CCD, Panasonic) was positioned approximately four feet above the center of the pool and video recording equipment (a PC with ULead Video Studio, Corel) was located 1.5–2.5 m from the pool behind the curtains surrounding the pool. Transfer cages were kept in a biocontainment hood on an opposite wall ∼ 4.5 m from the pool.

Escape training was performed in six blocks of three trials on a single training day (massed training), with a 15–30 min inter-block interval. The goal quadrant remained stationary throughout training but was varied across testing cohorts. Four starting locations, offset from the platform locations by 45°, were equally spaced around the pool (Figure 3). The starting location for each trial was chosen pseudo-randomly maintaining roughly equal representation of each starting location. Just prior to training, each rat was positioned to climb onto the escape platform three times from different directions. The first block of training trials immediately followed climbing practice. For each training trial, the animal was allowed a maximum of 1 min to find the platform. At the end of each trial, a fifteen second latency was imposed for the rat to spend on the platform. If the rat was unable to find the platform after 1 min, it was gently guided to the platform where it would remain for 15 s. After the sixth training block, an immediate probe trial (IMM) was performed in which the platform was removed, and the rat was allowed to explore the maze for 1 min. The platform was then replaced, and three more refresher trials were performed (not included in data analyses). Twenty-four hours after the IMM probe, the rat was placed in the MWM for a second, 1-min probe trial (24-HR probe).

### Tissue Collection

Following anesthesia (Isoflurane, >5% vapor) and before perfusion, adrenal and thymus glands were removed, placed on filter papers, and individually weighed. The animal was then perfused with 4% paraformaldehyde, the headcap was gently removed, and the brain was extracted.

### Corticosterone Analysis

Blood was collected 30 min after completion of the 24-HR probe either from decapitation during fresh tissue collection or by nicking the renal artery prior to perfusion. This time point was chosen because prior studies have shown that, while the exact time for CORT levels to peak following an acute stressor may vary somewhat, CORT remains significantly elevated at 30 min (Kim et al., 1998; Zimomra et al., 2011; Gil-Lozano et al., 2013). One half of each litter had blood collected from the renal artery prior to perfusion, and the other half from the trunk post decapitation. Blood was immediately centrifuged at 10,000 rpm at 4°C for 5 min. Plasma was then poured off into fresh Eppendorf tubes and stored at −20°C until assayed with a corticosterone ELISA kit (Enzo life sciences ADI-900-097, Farmingdale, NY).

### Statistical Analyses

For each training trial in the water maze, latency to escape and path length were measured. For each probe trial, the amount of time spent in each quadrant and distance to platform center (DtP, linear distance between the nose of the animal and the platform center sampled at 1 Hz) were analyzed (Figure 3). Proximity analyses like DtP have been shown to better separate learning impaired from normal subjects (Maei et al., 2009; Pereira and Burwell, 2015; Kapadia et al., 2016).

Repeated measures ANOVA were applied to the escape learning curves (repeating across trial blocks) and DtP measurement during the probe trials (repeating across seconds). None of the data passed Mauchly’s sphericity test, so the Greenhouse-Geisser correction was applied. Chi-squared tests were used to identify likelihood trends for categorical data, as determined by quadrant preference for both the IMM and 24-HR probes. ANOVAs were used to identify stress schedule effects on plasma CORT levels and tissue weights followed by Tukey *post hoc* tests. Data were averaged within litter prior to analysis or data were averaged within animal and litter number was added as a random factor, where appropriate.

## Results

### MD on a P2–11, but Not RAN Schedule, Impaired Escape Learning

**FIGURE 4:**
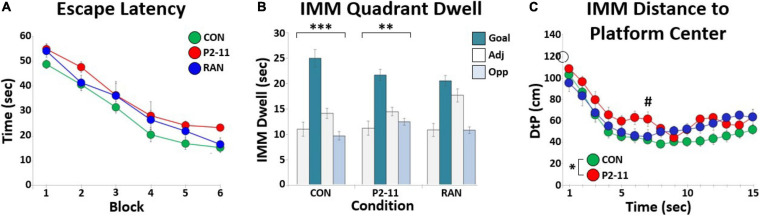
Summary of MD effects on escape learning in the MWM. **(A)** Litter mean escape latency in seconds for each training block across MD conditions. Sample sizes: CON = 11; P2–11 = 4; RAN = 6. **(B)** Bar graphs showing litter mean quadrant dwell time for each MD condition during the IMM probe trial. Asterisks denote significant Chi Square tests for quadrant (^∗∗∗^*p* < 0.001, ^∗∗^*p* < 0.01). **(C)** Each point marks the litter’s average distance between the animal and the platform location (DtP) across the first fifteen seconds of the IMM probe for each MD condition. The asterisk denotes significant RMANOVA main effect of MD condition (*p* < 0.05). The pound symbol (#) denotes significant Tukey effect of MD condition (*p* < 0.05). Sample sizes: CON = 11; P2–11 = 4; RAN = 6. The open circle on the *Y*-axis marks the starting location distance for all animals (120 cm).

A total of 21 litters were used for this study (P2–11: 4; RAN: 6; CON: 11). Figure 4A shows mean escape latency for each MD condition at each block of training. Litter mean escape latency decreased significantly across training blocks [*F*(2.606, 46.902) = 79.634, *p* < 0.001; Greenhouse-Geisser correction for non-sphericity], and there was no significant main effect of MD condition [*F*(2) = 2.789, *p* = 0.088]. There was no difference in escape learning between groups, suggesting no impairment in escape learning or performance selective to condition.

### MD on a P2–11, but Not RAN Schedule, Impaired the Initial Approach to the Platform Location During the IMM Probe Trial

For the IMM probe trial performed on the same day as the training trials, mean dwell time per quadrant was calculated for each litter’s MD group (Figure 4B). Chi Square tests and distribution shape indicated a goal quadrant bias for CON and P2–11 groups; CON [χ^2^(3, 11) = 33.000, *p* < 0.0001], RAN [χ^2^(3, 6) = 7.333, *p* = 0.0620], P2–11 [χ^2^(3, 4) = 12.000, *p* < 0.01]. We also analyzed the litter’s distance to the platform location (DtP) at each second for the first 15 s of the IMM probe trial to examine platform approach behavior. This measure has been compared to other measures of spatial accuracy and shown to be a more powerful tool in separating groups with varying spatial abilities (Maei et al., 2009; Pereira and Burwell, 2015; Kapadia et al., 2016). A two-way repeated measures ANOVA of litter means revealed main effects of sample time [*F*(2.653, 47.749) = 31.326, *p* < 0.001; Greenhouse-Geisser correction] and MD condition [*F*(2, 18) = 4.009, *p* < 0.05] (Figure 4C). Only the litters in the P2–11 group were consistently farther from the platform location than the CON group [Tukey: CON and P2–11: *p* < 0.05; *p* < 0.05 for second 7, *p* < 0.06 for seconds 6, 11; RAN and CON: *p* = 0.303; RAN and P2: *p* = 0.920]. These DtP results are not a function of group differences in swim speeds [cm/s across first 15 s ANOVA with litter as a random factor: CON, 13.17 ± 0.52; P2–11, 14.84 ± 0.70; RAN, 12.43 ± 0.89; *F*(2) = 2.261, *p* = 0.109]. Overall, these findings indicate that although P2–11 animals spent a similar amount of time in the goal quadrant as the CON and RAN animals, the P2–11 group was less direct in its initial approach to the platform location.

### Long-Term Spatial Memory Was Not Affected by MD

**FIGURE 5:**
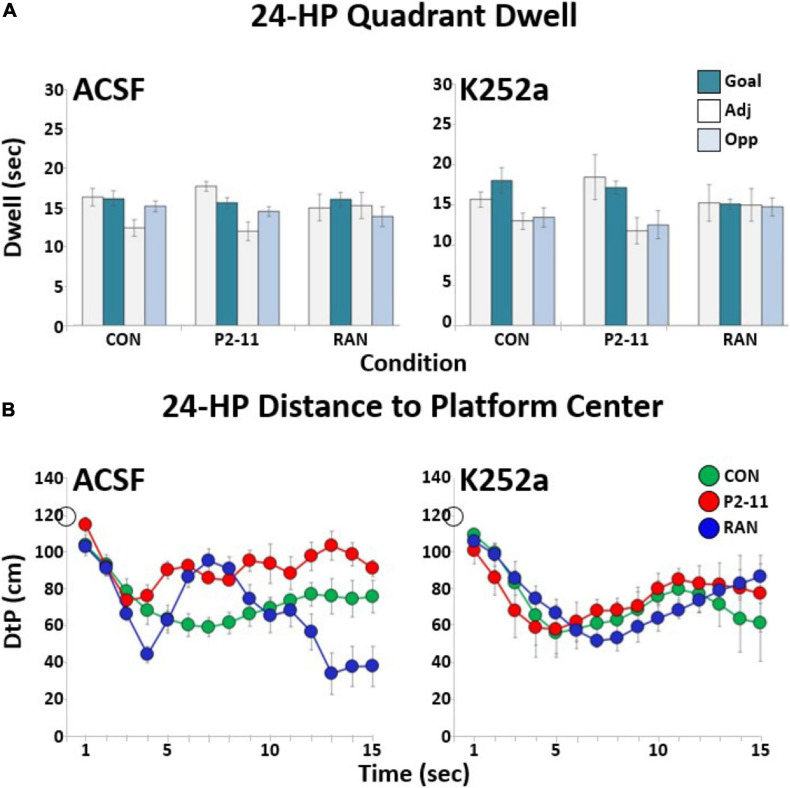
Summary of MD effects on spatial memory in the 24 h probe trial. **(A)** Quadrant dwell time distributions of litter means across MD and drug conditions. **(B)** Litter distance to platform location across MD and drug conditions. Sample sizes: ACSF (CON = 10; P2–11 = 4; RAN = 6); K252a (CON = 10; P2–11 = 3; RAN = 5). The open circle on the *Y*-axis marks the starting location distance for all animals (120 cm).

Spatial memory was assessed in the 24-HR probe trial. Typical of massed training protocols (Commins et al., 2003), some forgetting occurred in all groups and there was no clear goal quadrant bias (no significant Chi Square results) for any MD group following delivery of ACSF (Figure 5A, left side) or K252A (Figure 5A, right side). Two-way repeated measures ANOVAs on DtP of litter means indicated no main effect of sample time [*F*(5.086, 43.229) = 0.423 *p* = 0.866], or of MD condition [*F*(2) = 1.162, *p* = 0.336] following ACSF delivery (Figure 5B, left side) or K252a [time—*F*(5.926, 44.448) = 0.826, *p* < 0.555; MD condition—*F*(2) = 0.045, *p* = 0.956] (Figure 5B, right side).

### CORT Levels Following MWM Testing Were Unaffected by MD

**FIGURE 6:**
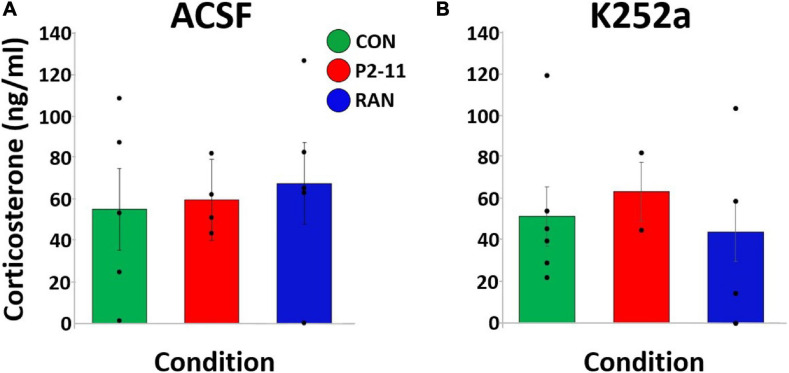
Mean plasma CORT levels collected after the 24-HR probe trial across MD and drug conditions. Dots represent individual litter means. **(A)** CORT levels for animals treated with ACSF before the 24-HR probe trial. **(B)** CORT levels for animals treated with K252a before the 24-HR probe trial. Sample sizes: ACSF: CON = 4, P2–11 = 4, RAN = 5; K252a: CON = 6, P2–11 = 2, RAN = 4.

We first compared CORT levels between collection procedures (trunk blood following decapitation or by nicking the renal artery). There were no differences in mean CORT level between different blood collection methods [*F*(1, 49) = 0.146, *p* = 0.704]. Therefore, values from each collection method were combined and averaged within experimental group. A two-way ANOVA of litter means revealed no main effect of MD condition [*F*(2) = 0.082, *p* = 0.921] or drug treatment [*F*(1) = 0.233, *p* = 0.635] on CORT level [*F*(2) = 0.254, *p* = 0.778; Figures 6A,B].

### Body Weight Tended to Be Reduced at Puberty Following P2–11 MD, but Adrenal and Thymus Weights Were Unaltered

**FIGURE 7:**
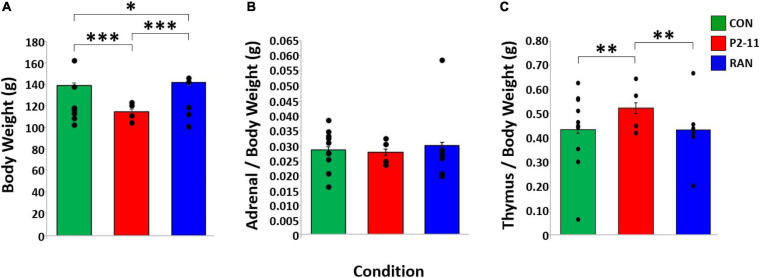
Body and tissue weights across MD groups. Dots represent individual litter means. **(A)** Body weights at time of surgery (P30) compared across MD condition with litter as a random factor. Body weight sample sizes: CON = 58; P2–11 = 24; RAN = 33. **(B)** Adrenal weight normalized by body weight across MD condition. Adrenal weight sample sizes: CON = 58; P2–11 = 24; RAN = 33. **(C)** Thymus weight normalized by body weight across MD condition. Thymus weight sample sizes CON = 58; P2–11 = 24; RAN = 33; RAN = 10. Asterisks denote significance (^∗∗∗^*p* < 0.001, ^∗∗^*p* < 0.01, ^∗^*p* < 0.05).

Body weight at time of surgery was different between MD conditions when litter was treated as a random factor [*F*(2, 95) = 21.920, *p* < 0.001] CON (*M* = 140.60 g) and RAN (*M* = 142.23 g) animals weighed more than P2–11 (*M* = 114.99 g) animals (Tukey; *p* < 0.001), suggesting less robust physical development after P2–11 MD and more robust development after RAN MD (Figure 7A). There was no effect of MD condition on normalized adrenal gland when litter was treated as a random factor [*F*(2, 95) = 0.424, *p* = 0.656)] (Figure 7B) and there was an effect of MD on normalized thymus gland weight when litter was treated as a random factor [*F*(2, 95) = 4.660, *p* = 0.012) (Figure 7C). P2–11 thymus was heavier than CON and RAN (Tukey; P2–11 to CON, *p* < 0.05; P2–11 to RAN, *p* < 0.01).

## Discussion

Prior rodent studies of early life stress incorporated MD schedules of varying types and assays performed in midlife (Plotsky and Meaney, 1993; van Oers, 1998; Weaver et al., 2007; Daskalakis et al., 2011; Suri et al., 2013). This study aimed to differentiate the effects of two schedules of MD, daily (P2–11) and random randomly spaced sessions (RAN), on spatial learning and memory ability in pubertal rats. Based on quadrant dwell time for the entire probe trial, RAN animals appear to be less persistent in their goal location search compared to CON and P2–11 subjects. However, this traditional measure is subject to off-target searching later in the probe trial. Compared to CON and RAN animals, the P2–11 schedule produced an impairment in the initial trajectory and proximity to the goal location. These measures have been shown to be better separators of spatial learning (IMM probe) and memory (24-HR probe) abilities across testing groups in MWM probe trials (Maei et al., 2009; Pereira and Burwell, 2015; Kapadia et al., 2016). There was a trend for an effect in the RAN group (*p* = 0.06), but the initial trajectory for this group aligns almost exactly with the CON group. Thus, the platform approach deficit observed when MD occurs from P2 to P11 is due to more frequent MD episodes during the first two postnatal weeks or the daily nature of the MD. Conversely, the lack of an approach deficit in the RAN group may have resulted from less intense MD during the first two postnatal weeks or the random spacing of the MD. There was no effect of MD schedule or K252A on 24-HR probe performance suggesting no impact neonatal stress on spatial memory or BDNF/TrKB signaling at this life stage. Future experiments will need to address the remaining outstanding variable of total age range of MD bouts (10 versus 20 days) and employ additional cognitive measures to better define the degree (spaced training in the MWM) and specificity (non-spatial learning and memory tasks) of the spatial learning impairment following daily MD. This study suffered somewhat from a low N that was mitigated in part by the reduced variability gained by averaging pup data within a litter. Larger litter numbers per group would add more statistical power and enable more concrete conclusions.

P2–11 animals weighed significantly less than RAN, but not CON subjects. These results suggest that growth reduction due to daily neonatal stress may begin at or near puberty. When normalized by body weight, the wet weights of the adrenal and thymus glands were unaltered, supporting the idea that the adrenal hyperplasia and thymus atrophy observed in adulthood emerge after puberty (Margarinos and McEwen, 1995; Ulrich-Lai et al., 2006).

**FIGURE 8:**
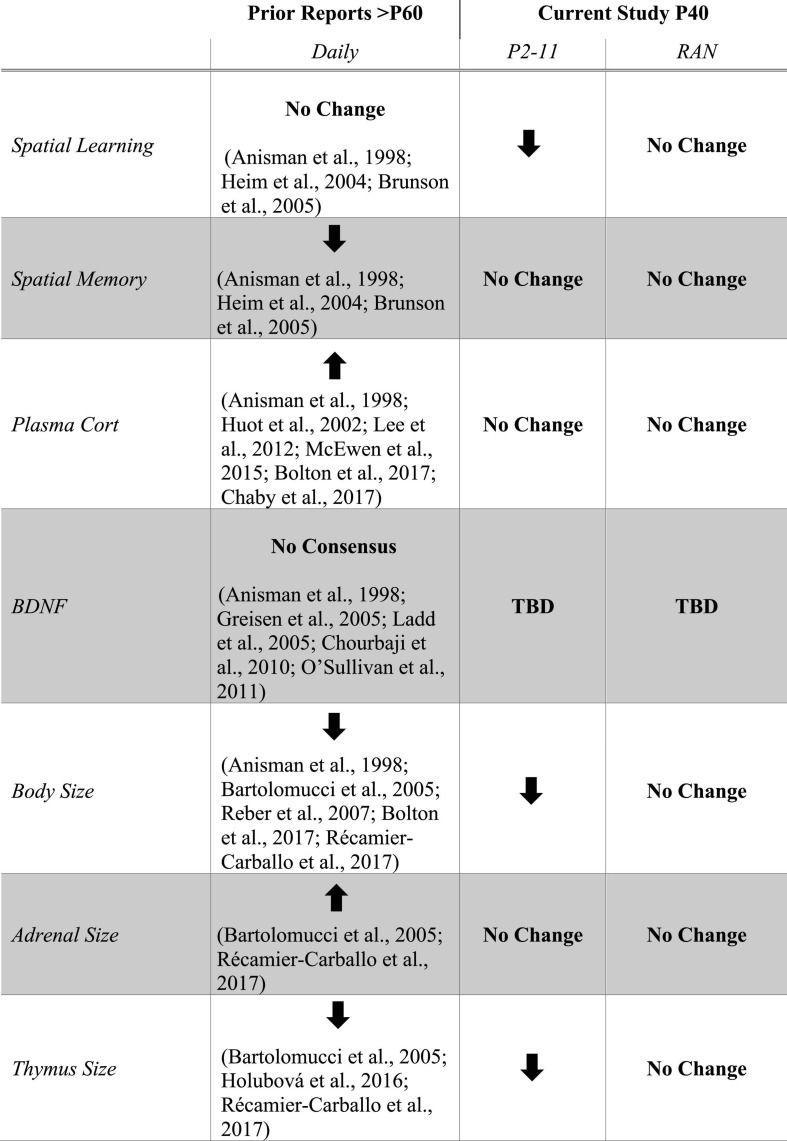
Table listing results from prior studies of MD with daily separation side-by-side with the current study. The Prior Reports column shows the consensus of research studies utilizing a daily (*Daily*) separation schedule with assays performed in adulthood (Anisman et al., 1998; Huot et al., 2002; Heim et al., 2004; Bartolomucci et al., 2005; Brunson et al., 2005; Greisen et al., 2005; Ladd et al., 2005; Reber et al., 2007; Chourbaji et al., 2010; O’Sullivan et al., 2011; Lee et al., 2012; McEwen et al., 2015; Holubová et al., 2016; Bolton et al., 2017; Chaby et al., 2017; Récamier-Carballo et al., 2017). The Current Study columns summarize the results from the P2–11 and RAN separation schedules. Arrows indicate direction of change in each parameter relative to non-stressed controls. No Change indicates that there is no difference between stressed and non-stressed controls. No Consensus indicates that there is no clear agreement in the literature. TBD indicates “to be determined.”

The effects of early life stress on spatial learning are schedule-dependent and may differ when viewed at puberty (this study) versus adulthood (prior studies) (Figure 8). While caution should be taken when comparing across life stages (because age of testing is not the only difference between studies), the current findings support the need for studies directly comparing across various life stages. Only after the P2–11 separation schedule was spatial learning impaired. Due to the massed training protocol, all groups showed forgetting. A lack of performance differences across conditions suggests that neither of the MD conditions was able to improve long-term memory following massed training. While not performed within the same study, this is in sharp contrast to effects observed in adulthood (Brunson, 2005; Bolton et al., 2017; Chaby et al., 2017). Long-term memory may be more completely understood after a spaced training paradigm that enhances long-term memories. These findings show that the effects of different MD schedules can be differentiated at puberty and support the notion that more consistent early life stress is required to produce cognitive dysfunction at life stages earlier than adulthood. However, it is not clear if the behavioral outcome after MD from P2 to P11 is due to the full 10 consecutive days of MD or some critical number of separation bouts during the first postnatal week (Lehmann and Feldon, 2000; Litvin et al., 2010; Tractenberg et al., 2016; Borges-Aguiar et al., 2018; Orso et al., 2019).

Inclusion of only male subjects polarizes the CORT data as females show a differential CORT response to early life stress at puberty (Romeo et al., 2004; Hodes and Epperson, 2019). Specifically, intact females exhibit an extended CORT response whereas males more rapidly return to baseline after an acute stressor. This manifests in the males showing greater resilience to acute stress during adolescence and adulthood while females exhibiting more persistent, negative behavioral effects (Bourke and Neigh, 2011). Moreover, gender differences in MD responsiveness (Weaver et al., 2007; Oreland et al., 2009) warrant replication of this study on female rats.

Baseline CORT is highly variable amongst laboratory rats housed under the same conditions (García and Armario, 2001). By clamping this natural variability, via adrenalectomy with CORT replacement, or by employing within-animal CORT comparisons before and after MWM testing, effects on plasma CORT might have been revealed. As well, collecting blood at multiple time points following maze exposure to analyze CORT might have revealed more information about the changes in CORT over time and the reason for low CORT levels in this particular study. In addition, many studies have shown that handling of pups during their juvenile period, which we did, may result in decreased anxiety and a more positive reaction to later stressors (Chapillon et al., 2002), including altering of the corticosterone response (Levine et al., 1967; Meaney et al., 1985; Levine, 2000). These factors may have reduced the overall impact of MD in our study.

Finally, maternal effects due to the use of first litters instead of those from seasoned dams may have also influenced the outcome (Pawluski et al., 2009). Significant increases in CORT are found from P1 in primi- versus multiparous females (Grajeda and Pérez-Escamilla, 2002; Pawluski et al., 2009) and is correlated with increased licking and grooming in rats (Pawluski et al., 2009), though the differences in CORT and maternal care between primi- and multiparous females disappears by P14. It may be informative to further investigate connections between BDNF and CORT in regulating the progression of cognitive alterations following early life stress by measuring BDNF and CORT levels at numerous timepoints leading up to puberty.

One of the important aspects of this study is the identification of a uniquely timed stressor and its effects on the adult stress response. Most studies that look at the effects of MD focus on the first two postnatal weeks rather than the entire juvenile period (Wang et al., 2020). Some studies examine manipulations occurring daily for the first three postnatal weeks (Shimada et al., 1990; Meerlo et al., 1999; Pryce et al., 2001, 2003; Kosten et al., 2006). One study of maternal deprivation through P21 reported enhanced spatial learning in the MWM when deprived subjects were tested at 3–6 months of age (Pryce et al., 2003). Given variations in adult behavior based on frequency of MD bouts, total age span of MD bouts, and age of testing, larger studies directly comparing across these variables are needed to gain a more complete perspective on the lifespan impacts of MD. Moreover, while the relevance of animal studies to human MD equivalents is clear (Bolton et al., 2017), this field would benefit from early life stress models that more closely approximate human development. Randomized MD may provide greater insight into the lasting effects of more persistent early life stress occurring in an unpredictable manner, which may be more akin to human early life stressors.

## Conclusion

This study has illuminated effects of daily MD on stress biology and cognitive ability at a younger age than typically studied. Additional experiments at varying developmental stages are necessary to fully understand the trajectory of the cognitive and health impacts of early life stress. Currently, the spatial learning deficit at puberty may serve as a behavioral marker of future memory failure that can be assessed non-invasively and inexpensively at the human level. Testing of other types of learning and memory might reveal additional markers for impending memory loss and possibly provide a novel platform for prophylaxis aimed at averting not only cognitive dysfunction later in life but also the overwhelming cost of lifetime treatment for childhood abuse. For all individuals identified in a given year, the total cost to society is estimated to run upward of $100 billion dollars per year (Fang et al., 2012). Thus, for both quality of life and economic reasons, it is imperative that we further identify the underlying mechanisms of early life stress that leads to adult disease.

## Data Availability Statement

The raw data supporting the conclusions of this article will be made available by the authors without undue reservation.

## Ethics Statement

The animal study was reviewed and approved by the Institutional Animal Care and Use Committee, George Mason University.

## Author Contributions

ES and TD designed the study. ES, DM, SS-B, NL, CL, JE, and KB collected the data. ES analyzed the data. ES and TD co-wrote the manuscript. ES, DM, SS-B, NL, CL, JE, KB, and TD revised the manuscript. All authors contributed to the article and approved the submitted version.

## Conflict of Interest

The authors declare that the research was conducted in the absence of any commercial or financial relationships that could be construed as a potential conflict of interest.
